# Tension pneumomediastinum and diffuse subcutaneous emphysema with severe acute respiratory syndrome coronavirus 2 infection requiring operative management for impending airway collapse: A case report

**DOI:** 10.1002/ccr3.4656

**Published:** 2021-08-15

**Authors:** Kevin P. Lin, Christopher Stefaniak, Connor M. Bunch, Robert March, Mahmud Zamlut, Syed Raza, Walter Osorio, Josh Korzan, Jeffery Show, Nicolas Mjaess, Shivani Patel, Sufyan Zackariya, Ali Sualeh, Grant Wiarda, Hamid Al‐Fadhl, Anthony V. Thomas, Rashid Z. Khan, Laura Gillespie, Mark M. Walsh

**Affiliations:** ^1^ Indiana University School of Medicine South Bend Campus Notre Dame IN USA; ^2^ Department of Medical Affairs St. Joseph Regional Medical Center Mishawaka IN USA; ^3^ Department of Cardiothoracic Surgery St. Joseph Regional Medical Center Mishawaka IN USA; ^4^ Department of Intensive Care Medicine St. Joseph Regional Medical Center Mishawaka IN USA; ^5^ Departments of Emergency Medicine and Internal Medicine St. Joseph Regional Medical Center Mishawaka IN USA; ^6^ Department of Hematology Michiana Hematology Oncology Mishawaka IN USA; ^7^ Department of Quality Assurance and Performance Improvement St. Joseph Regional Medical Center Mishawaka IN USA

**Keywords:** case report, coronavirus, COVID‐19, drainage, mediastinal emphysema, pericardial window, pneumomediastinum, surgery

## Abstract

Tension pneumomediastinum is a rare complication of severe acute respiratory syndrome coronavirus 2 (SARS‐CoV‐2) infection that has increased in incidence with the novel coronavirus disease 2019 pandemic. Although traditionally managed with conservative measures, we present the indications and methods for the first operative management of tension pneumomediastinum with concomitant SARS‐CoV‐2 infection.

## Graphical abstract

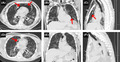

## 1 INTRODUCTION

Tension pneumomediastinum is a rare complication in patients infected with severe acute respiratory syndrome coronavirus 2 (SARS‐CoV‐2). Pneumomediastinum typically resolves with conservative management. Rarely, airway collapse, persistent hemodynamic instability, or progression to cardiac tamponade may require surgical intervention.^1^, ^2^ Described here is a unique case of SARS‐CoV‐2 infection requiring invasive ventilation that was later complicated by tension pneumomediastinum, which ultimately required operative anterior mediastinal and pericardial drainage. The scant literature on this phenomenon was reviewed, and we discuss the indications and methods for the first operative management of tension pneumomediastinum in a patient with the novel coronavirus disease 2019 (COVID‐19).^1^

## 2 CASE HISTORY

**FIGURE 1:**
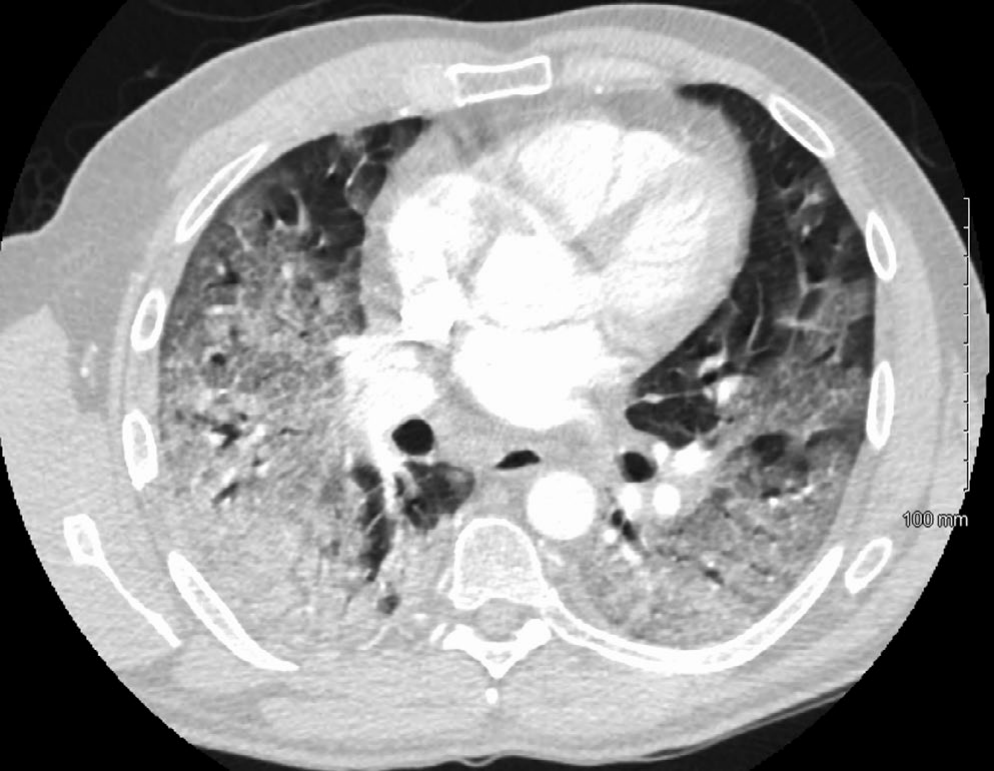
Computed axial tomography image in axial view of the lungs on admission demonstrated diffuse bilateral ground‐glass airspace disease consistent with COVID‐19 pneumonia

A 48‐year‐old male patient presented to the emergency department 6 days after onset of shortness of breath, cough, and chills. He tested positive for COVID‐19 5 days prior. On admission, vital signs showed blood pressure of 116/80 mmHg, temperature of 99.8 °F, heart rate of 109 beats per minute, respiratory rate of 40 breaths per minute, and oxygen saturation of 72% on room air. The patient was initially placed on 6 L/min oxygen by nasal cannula and escalated to 15 L/min. Serum laboratories on admission were significant for sodium of 126 mg/dl (standard range 135–145), potassium of 3.4 mg/dl (standard range 3.6–5.2), C‐reactive protein of 429 mg/L (standard range 0–10.0), lactate of 2.3 mMol/L (standard range 0.4–2.0), leukocytosis of 18,860 cells/L (standard range 4000–11,000), and D‐dimer of 1.52 mg/L (standard range 0–0.53). He was confirmed COVID‐19 positive by reverse transcriptase‐polymerase chain reaction of nasopharyngeal swab. Initial imaging revealed diffuse bilateral ground‐glass infiltrates above both lung fields, which was subsequently described as “tree‐in‐bud” acute respiratory distress syndrome (Figure 1). Four‐extremity Doppler ultrasound revealed a right upper extremity brachial vein deep vein thrombus (DVT), and the patient was placed on twice daily 1 mg/kg subcutaneous enoxaparin.

On the same day as admission, the patient continued to have tachycardia, tachypnea, and low oxygen saturation despite high flow oxygen by nasal cannula and required endotracheal intubation and mechanical ventilation for the next 48 h due to respiratory insufficiency. The patient self‐extubated on day 4 of hospitalization and remained tachypneic until the last 12 h of hospitalization. He was discharged on day 10 and prescribed apixaban 5 mg twice daily for upper extremity DVT therapy. The patient did not require oxygen supplementation at discharge.

Three days after discharge, the patient developed a sense of fullness in his neck. Five days after discharge, his family physician made a house call and noticed that the patient had resting tachycardia at 108 beats per minute, oxygen saturation of 93% on room air, and a heart rate that increased to 140 while walking in place for one minute. The patient had faint crackles over the right posterior lung field but no evidence of subcutaneous crepitus at this time.

**FIGURE 2:**
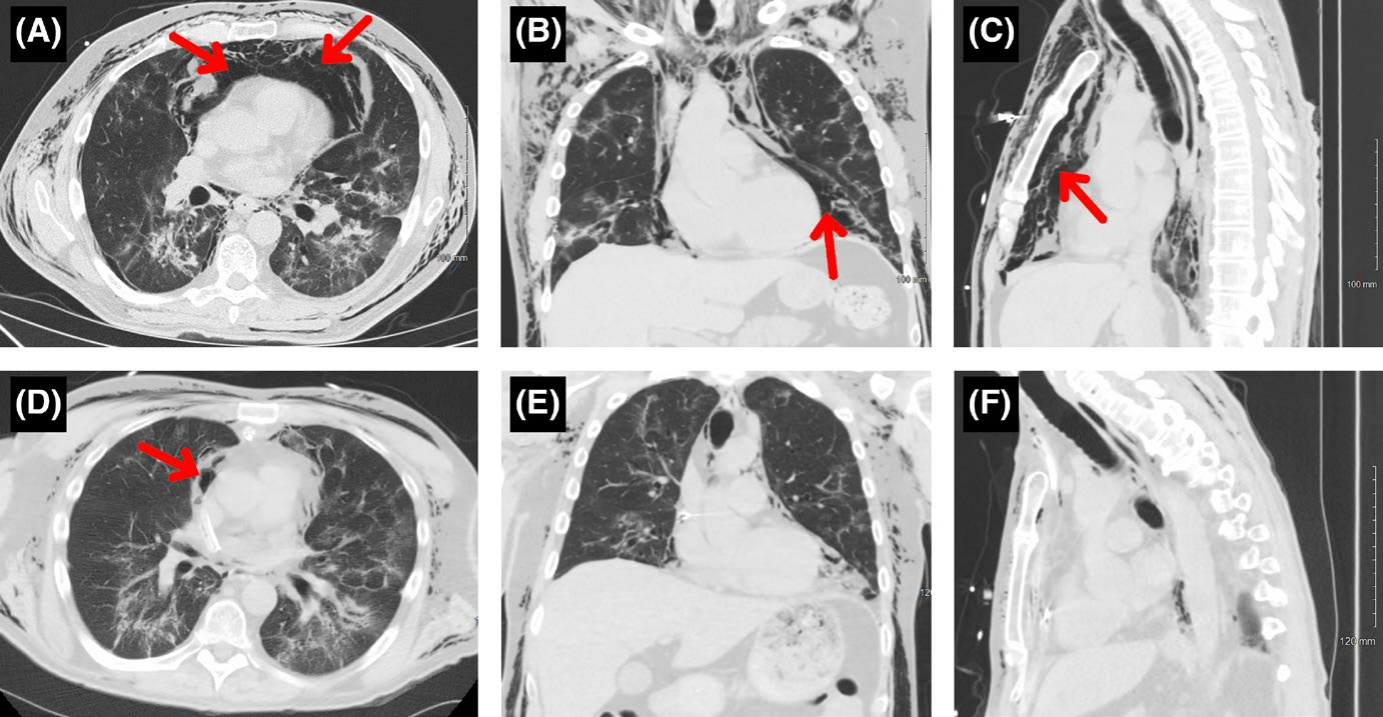
Computed axial tomography images of the lungs upon readmission in axial view (A), coronal view (B), and sagittal view (C) demonstrated the resolution of the bilateral infiltrates from prior hospitalization. However, the patient developed massive tension pneumomediastinum (indicated by red arrows). Subcutaneous air is also depicted throughout the thorax and inferior neck. There is also a small right‐sided pneumothorax visualized in panel A. Computed axial tomography images of the lungs 3 days post‐operative in axial view (D), coronal view (E), and sagittal view (F) demonstrated a marked reduction in pneumomediastinum and subcutaneous emphysema

On the seventh day following discharge and 2 days after the physician house call, the patient returned to the emergency department with increasing swelling in the neck and a sensation of ″crackling″ in his neck, chest, and scrotum. Vital signs included blood pressure of 120/70 mmHg, heart rate of 112 beats per minute, oxygen saturation of 94% on room air, respiratory rate of 28 breaths per minute, and temperature of 99.6 °F. Physical examination revealed increased swelling of the neck compared to 2 days prior, palpable crepitus in the neck, chest, and abdomen, and diminished breath sounds over both lung bases. There was no audible Hamman's crunch sign of a pericardial friction rub. A computed axial tomography (CT) scan revealed diffuse subcutaneous emphysema in the neck and chest with massive tension pneumomediastinum, but a marked improvement of the previous parenchymal infiltrates (Figure 2A–C)

The patient was admitted to the hospital for monitoring. 48 h later, the patient's subcutaneous emphysema spread to his arms as demonstrated by newly palpable crepitus from his axilla to the wrists. Additionally, there was significantly increased swelling and crepitus in the neck. The patient described difficulty with breathing due to a sense of local constriction in his upper airway with progressively increased stridor and increased pitch of his voice that caused great difficulty with speaking and breathing.

**FIGURE 3:**
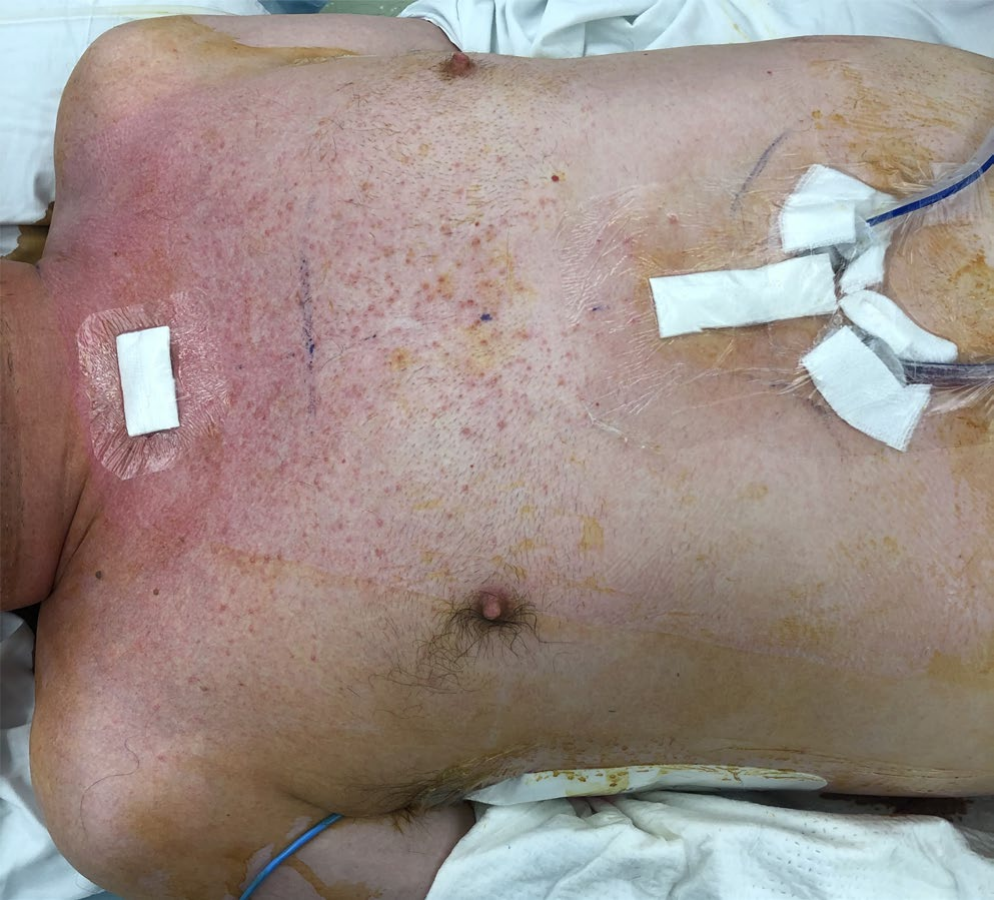
Post‐operative photograph depicting anterior mediastinal and pericardial window Blake drains (24 French) exiting through the subxiphoid region

With concern for impending airway obstruction, the patient was taken for emergency mediastinal drainage. He received a subxiphoid pericardial window, subxiphoid and suprasternal drainage of the pneumomediastinum, substernal dissection with a lighted scope, and laryngotracheobronchoscopy. A suprasternal notch transverse incision and dissection to the anterior mediastinum was also performed. A lighted balloon‐tipped endoscope was used to further develop the substernal space from the subxiphoid space up to the suprasternal notch. Neither pleural space was entered. The anterior mediastinum was completely decompressed, and a Blake drain (24 French) was placed and exited through a separate space in the subxiphoid region (Figure 3). A pericardial wound was then created in this space and was drained as well with a Blake drain (24 French). Fiberoptic bronchoscopy was then conducted to confirm that there were no injuries extending down to the major bronchi. A repeat CT scan taken 3 days postoperatively demonstrated a marked reduction in tension pneumomediastinum (Figure 2D–F). A repeat ultrasound of the upper extremity postoperatively demonstrated a complete disappearance of the brachial vein thrombus present only 2 weeks prior. The patient made an uneventful recovery with complete clinical and radiographic healing.

## 3 DISCUSSION

Tension pneumomediastinum is a rare but potentially lethal condition seen in critically ill patients. Traditionally, pneumomediastinum occurs in young patients with asthma. During an asthmatic attack, rapid breathing causes alveolar rupture into the lower‐pressure mediastinum. This condition is often harmless and resolves spontaneously as air is absorbed with time.^3^ On the other hand, tension pneumomediastinum can also occur due to prolonged mechanical ventilation, particularly in settings of high end‐expiratory pressure.^2^ Understandably so, this complication has seen an increase in incidence following the emergence of the COVID‐19 pandemic, as high end‐expiratory pressure ventilation has been utilized to a greater extent for management of COVID‐19–related respiratory distress.^1^ This form of pneumomediastinum is far more complicated and requires urgent intervention.

Tension pneumomediastinum is thought to occur in patients with COVID‐19 secondary to diffuse alveolar damage. The increased presence of diseased alveoli on the mediastinal surface allows for preferential rupture into the mediastinum due to the pressure gradient between the alveoli and the perivascular sheaths. Further spreading of the pulmonary interstitial emphysema into the mediastinum is subsequently known as the Macklin effect.^4^ In patients with COVID‐19, the diseased lung may create a one‐way valve at the mediastinal/pleural border, which can subsequently lead to air retention in the mediastinum. Increased pressure in the mediastinum can compress mediastinal contents. In particular, compression of the great vessels can lead to decreased venous return, hypotension with tachycardia, and potential cardiovascular collapse.^2^

Currently, management for tension pneumomediastinum in the COVID‐19 population has largely been conservative.^1^ Different approaches include reducing airway pressures and adjusting ventilator settings to allow for permissive hypercapnia in an effort to reduce pressure gradients across the mediastinal surface. These methods may be sufficient for the management of tension pneumomediastinum in stable COVID‐19 patients, but those who are unstable may require immediate surgical decompression. After review of the current literature, we describe the first case report of operative management for a massive tension pneumomediastinum secondary to COVID‐19.^1^ Of note, there was one previous report of tension pneumomediastinum secondary to COVID‐19 that resolved with bedside mediastinotomy via the Chamberlain procedure.^5^

In our patient with COVID‐19, a tension pneumomediastinum formed in the chest and neck, with subsequent spread to the arms bilaterally. The enlarging pneumomediastinum caused difficulty breathing and progressive dysphonia with an increased pitch in the tone of his voice. Due to impending airway obstruction, the patient was sent for emergent mediastinal drainage. Specifically, we created a subxiphoid pericardial window, employed subxiphoid and suprasternal drainage of the pneumomediastinum, and performed substernal dissection with lighted scope. The anterior mediastinum was decompressed with this surgical management, resulting in rapidly reduced swelling in the patient's neck, improvement of his voice, and disappearance of the crepitus with clinical and radiographic healing.

We describe the first operative management of massive tension pneumomediastinum secondary to SARS‐CoV‐2 infection. We used an operative technique that provided rapid decompression of unstable tension pneumomediastinum using a pericardial window and mediastinal drain. This case demonstrates that precipitous decline may occur in a patient with diseased lung parenchyma such as COVID‐19, and that our method may offer an effective operative solution for rapid decompression required for massive tension pneumomediastinum dissolution.

## CONFLICT OF INTEREST

The authors declare that they have no competing interests.

## AUTHOR CONTRIBUTIONS

KPL, CS, RM, RZ, RZK, LG, and MMW conceptualized the manuscript. KPL, CS, CMB, NM, SP, SZ, AS, GW, HA, and MMW reviewed the literature. All authors critically revised the manuscript and approved the final draft.

## ETHICS STATEMENT

This protocol development was approved by the Institutional Review Board of Saint Joseph Regional Medical Center at Mishawaka, IN.

## CONSENT

Informed consent was obtained from the patient to publish the case report.

## Data Availability

All relevant data are presented in the case. Any other data or inquiries about the case are available from the corresponding author upon request.
